# LIM-Domain-Binding Protein 1 Mediates Cell Proliferation and Drug Resistance in Colorectal Cancer

**DOI:** 10.3389/fsurg.2021.790380

**Published:** 2022-01-06

**Authors:** Mo Zhu, Baofei Jiang, Hao Zuo, Xiaopeng Wang, Hengfa Ge, Ziming Huang

**Affiliations:** ^1^Department of Gastrointestinal Surgery, The Affiliated Huaian No.1 People's Hospital of Nanjing Medical University, Huai'an, China; ^2^Department of Emergency Surgery, The Affiliated Huaian No.1 People's Hospital of Nanjing Medical University, Huai'an, China

**Keywords:** LIM-domain-binding protein 1, cell growth, drug sensitivity, oxaliplatin, colorectal cancer

## Abstract

**Objective:** It has been shown that LIM-domain-binding protein 1 (LDB1) is involved in the tumorigenesis of several cancers, but its function in colorectal cancer (CRC) has not been fully explained. This study is aimed to investigate whether LDB1 is involved in regulating the cell growth and drug sensitivity of CRC.

**Methods:** To analyze the protein expression of LDB1 in CRC tissues, western blot was used. KM plotter and UALCAN databases were used to predict the prognosis of CRC patients with low or high LDB1 expression. To do the correlation analysis in CRC tissues, GEPIA database was used. CCK-8 assay and xenograft models were used to evaluate the effects of LDB1 in CRC cell growth. An oxaliplatin-resistant cell line was constructed to evaluate the effect of LDB1 in drug sensitivity of CRC cells.

**Results:** Our current research confirmed that LDB1 was upregulated in CRC tumor tissues, and its elevation predicted a poor prognosis for CRC patients. LDB1 was also found positively correlated with CCNA1, BCL2 and BCLW, but negatively correlated with the pro-apoptotic signals (BID, BAX and BAK). Silence of LDB1 significantly inhibited CRC cell growth *in vitro*, and CRC cells with low expression of LDB1 had a lower tumorigenesis rate in tumor-bearing nude mice. Our experiments also showed that LDB1 silence enhanced the anti-tumor activity of oxaliplatin in CRC cells. The expression of LDB1 was also found increased in oxaliplatin-resistant CRC cell lines, and silence of LDB1 partly restored the antitumor effect of oxaliplatin in an oxaliplatin-resistant CRC cell line.

**Conclusion:** Our current results revealed the roles of LDB1 in the growth and drug resistance of CRC cells, and may provide the new theoretical support for LDB1 as a potential target for the treatment of CRC in the future.

## Introduction

Colorectal cancer (CRC) is a common malignant cancer of the digestive system, which mainly occurs in the rectum and the junction of rectum and sigmoid colon, accounting for about 60% (1). What's more, the incidence of CRC is second only to gastric cancer and esophageal cancer, and the incidence rate is still rising (2). At present, the important methods for the treatment of CRC mainly include surgery, chemotherapy, radiotherapy and targeted therapy (3). Among them, 5-FU, platinum, irinotecan and capecitabine are the traditional chemotherapeutic drugs for CRC (4). However, traditional chemotherapeutic drugs are easy to cause drug resistance and display side effects (5). For example, it is easy for CRC cells to acquire resistance to the platinum drugs and develop resistance (6). Therefore, more specific therapeutic methods are urgently needed. In recent years, great progress has been made in the treatment and diagnosis of CRC (7, 8), however, the further improvement of the survival rate of CRC is still limited. Given these, identifying early diagnosis and prognostic markers of CRC are of great significance for guiding treatment and improving the prognosis of patients.

LIM-domain-binding protein 1 (LDB1), also known as NLI or CLIM2, is a multi-adaptor protein that is closely involved in carcinogenesis, hematopoiesis, cardiogenesis, neurogenesis, etc (9). So far, some papers have reported the functions and roles of LDB1 in several tumors. For example, in the proto-oncogene LMO2-induced T-cell acute lymphoblastic leukemia, LMO2 along with LDB1, b-HLH and GATA formed a multi-subunit transcription complex, which was required for the oncogenic transformation of immature thymocytes (10). Another paper also confirmed that LDB1 enhanced the stability of direct and indirect oncoprotein partners in leukemia, such as LMO2 (11). In squamous cell carcinoma of the head and neck, loss of LDB1 significantly inhibited growth and vascularization of xenografted tumors (12). And our previous study also reported that the ubiquitin ligase RNF38 regulated CRC cell growth by degrading LDB1, but the function of LDB1 was not studied in depth (13).

In the current study, we aimed to further elucidate the function of LDB1 in the growth and drug resistance of CRC. Our results in this paper confirmed that LDB1 was upregulated in CRC, and its knockdown significantly inhibited CRC cell growth *in vitro* and suppressed tumor growth *in vivo*. Moreover, we found that knockdown of LDB1 significantly enhanced the anti-tumor activity of oxaliplatin in CRC cells, and LDB1 knockdown also partly restored the antitumor effect of oxaliplatin in an oxaliplatin-resistant CRC cell line. Our paper may provide a theoretical basis for the further use of LDB1 as a drug target for the treatment of CRC.

## Materials and Methods

### Cells, Chemicals and Patient Samples

HCT116, DLD1 and HEK293T cell lines were purchased from ATCC, Manassas, Virginia, USA. Cells were cultured in Dulbecco's Modified Eagle Medium (Meilunbio, Dalian, China; Cat. MA0212-1) with 10% fetal bovine serum (Gibco, California, USA; Cat. 10099-141). The cells were passaged by 0.25% Trypsin-EDTA (Meilunbio, Dalian, China; Cat. MB4376) digestion. Oxaliplatin was purchased from Sigma-Aldrich, St. Louis, Missouri, USA (Cat. 09512). Oxaliplatin-resistant DLD1 cells (DLD1/OXA) were established by continuous culture for 3 months in the selection of oxaliplatin as described previously (14). Twelve pairs of CRC paracancerous and tumor tissues were obtained from The Affiliated Huaian No.1 People's Hospital of Nanjing Medical University. The collection and use of the CRC tissues were approved by the Review and Ethics committee of The Affiliated Huaian No.1 People's Hospital of Nanjing Medical University, and all the patients provided written informed consent.

### Western Blot

Cells were lysed by RIPA lysis (Beyotime; Cat. P0013B) with protease inhibitor cocktail (Bimake.cn; Cat. B14012). Total protein was obtained by centrifugation at 4°C (12,000 rpm, 30 min), and protein concentration was quantified by BCA assay (Beyotime; Cat. P0010). 30 μg total protein was loaded for SDS-PAGE analysis, and blotted onto PVDF membranes (Millipore; Cat. ISEQ00010). The anti-LDB1 (Cat. sc-514035) and GAPDH (Cat. sc-47724) primary antibodies were obtained from Santa Cruz Biotechnology, CA, USA. The specific experimental steps were shown in the previous published article (15).

### Bioinformatics Analysis

To analyze the role of LDB1 in the prognosis of patients with CRC, Kaplan-Meier Plotter based on the Pan-cancer RNA-seq database was used online (http://kmplot.com). In the process of analysis, rectum adenocarcinoma (*n* = 165) was chosen, and other options were set to default values (auto select best cutoff was checked and the cutoff value used in analysis was 926). In addition, the effect of LDB1 expression level on colon adenocarcinoma (COAD) patients' survival was also analyzed online by UALCAN (http://ualcan.path.uab.edu/).

The correlation analysis between LDB1 and CCNA1, BCL2, BCLW, BID, BAX or BAK in COAD was analyzed by GEPIA database online (http://gepia2.cancer-pku.cn/#correlation). The expression datasets used were as follows: COAD Tumor, COAD Normal, Colon-Sigmoid.

### Quantitative Real-Time PCR

Total RNA from CRC cells was extracted by using Total RNA Extraction Reagent (Vazyme, Nanjing, China; Cat. R401-01), and cDNA was reversely transcribed by using HiScript^®^ II Q Select RT SuperMix (Vazyme; Cat. R233-01). Quantitative real-time PCR (qRT-PCR) was carried out by using ChamQ^TM^ Universal SYBR^®^ qPCR Master Mix (Vazyme; Cat. Q711-02) on a ABI 7500 PCR System (Applied Biosystems, CA, USA). The primers used for qRT-PCR were listed as in Table 1.

**Table 1:** The primers used in the study.

**Gene name**	**The sequences of primers**
LDB1	forward, 5′-GCTGTGCCTGTCCTGGTT-3′
	reverse, 5′-GCCCACATCCCTATCCAG-3′
CCNA1	forward, 5′-ACTGCTGCTATGCTGTTA-3′
	reverse, 5′-TGGTGTAGGTATCATCTGTAAT-3′
BCL2	forward, 5′-ATGACTGAGTACCTGAACC-3′
	reverse, 5′-AGACAGCCAGGAGAAATC-3′
BCLW	forward, 5′-GAAGGGTTATGTCTGTGGAGC-3′
	reverse, 5′-CCTGGGTGAAGCGTTGTT-3′
GAPDH	forward, 5′-GCACCGTCAAGGCTGAGAAC-3′
	reverse, 5′-TGGTGAAGACGCCAGTGGA-3′

### ShRNAs, Lentivirus Packaging and Infection

Control (sc-108060) and LDB1-directed lentiviral shRNAs (sc-35072-SH) were purchased from Santa Cruz Biotechnology, CA, USA. Lentiviruses were made by transfecting HEK293T cells with shRNAs and packaging mix (Santa Cruz Biotechnology) by using Lipofectamine^®^ 2000 (Invitrogen; Cat. 11668019) according to the manufacturer's instructions. Seventy-two hours later, the viral supernatant was collected and concentrated. CRC cells were incubated with viral particles in the presence of 6 μg/ml polybrene (Beyotime; Cat. C0351), and infected cells were selected in 2 μg/ml puromycin (Beyotime; Cat. ST551).

### Cell Growth and Survival

To detect the effect of silencing LDB1 on the growth of CRC cells, CRC cells infected with control or LDB1 shRNA-derived lentivirus were cultured for 0, 1, 2, 3, or 4 days respectively, followed by CCK-8 assay by using CCK-8 Cell Counting Kit (Vazyme; Cat. A311-01/02) according to the instructions. In order to detect the effect of oxaliplatin on the survival of CRC cells, CRC cells were incubated with increasing concentrations of oxaliplatin (0, 10, 20, 40 or 60 μM) for 48 hours, followed by CCK-8 assay.

### Tumor Xenograft Models

Female BALB/c nude mice (four to 6 weeks) from Institute of model animals of Nanjing University (Nanjing, China) were used for animal study. DLD1 cells (5 × 10^6^ cells) infected with control or LDB1 shRNA-derived lentivirus were subcutaneously inoculated into the right flanks of nude mice (five mice/group). When tumors were palpable, tumor volume was monitored every 3 days by using vernier caliper. After the animal experiment, tumors were excised, weighed and taken photos. The animal study was approved by the Review and Ethics committee of The Affiliated Huaian No.1 People's Hospital of Nanjing Medical University.

### Statistical Analysis

The results were presented as mean ± SD. Students's *t*-test or ANOVA followed by Tukey's post hoc test was used to compare differences between groups. *P* < 0.05 showed significant difference in this study.

## Results

### LDB1 Is Upregulated and Predicts a Poor Prognosis in Colorectal Cancer

**Figure 1:**
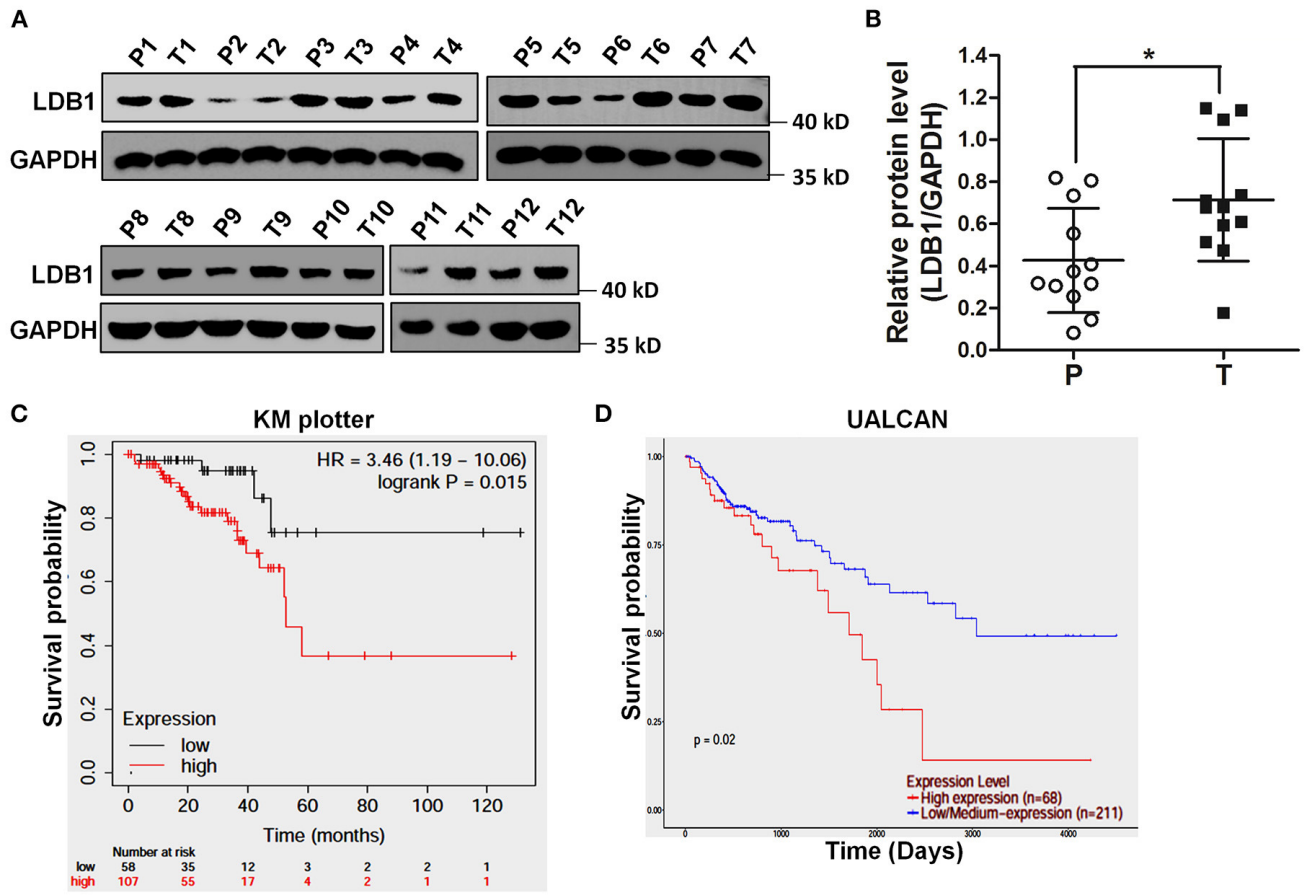
The expression level of LDB1 in colorectal cancer and its effects on the prognosis of patients. **(A,B)**. Twelve pairs of paracancerous (P) and tumor (T) tissues from colorectal cancer (CRC) were lysed for western blot to detect the expression of LDB1. GAPDH was used as a loading control **(A)**. The optical density analysis was also carried out **(B)**. **(C)** Effect of LDB1 expression level on rectum adenocarcinoma patient survival was analyzed online by KM plotter. **(D)** Effect of LDB1 expression level on colon adenocarcinoma patient survival was analyzed online by UALCAN. *P < 0.05.

To confirm the expression levels of LDB1 in CRC, twelve pairs of CRC paracancerous and tumor tissues were obtained and prepared for western blot analysis. As shown in Figures 1A,B, the results of western blot showed that LDB1 was upregulated in tumor tissues of CRC compared with the paracancerous tissues. Specifically, 8 out of 12 pairs of tissues (66.7%) showed markedly upregulated LDB1 expression in tumor tissues. And then, we used KM Plotter and UALCAN to assess the effect of LDB1 on overall survival of CRC patients. As shown in Figures 1C,D, the results showed that the overall survival rate of CRC patients with high expression of LDB1 was significantly lower than that patients with low LDB1 expression. Our present results further confirmed that LDB1 was a poor prognosis for CRC patients.

### Knockdown of LDB1 Inhibits Cell Growth in Colorectal Cancer Cells, and Suppresses Tumor Growth in Nude Mice

**Figure 2:**
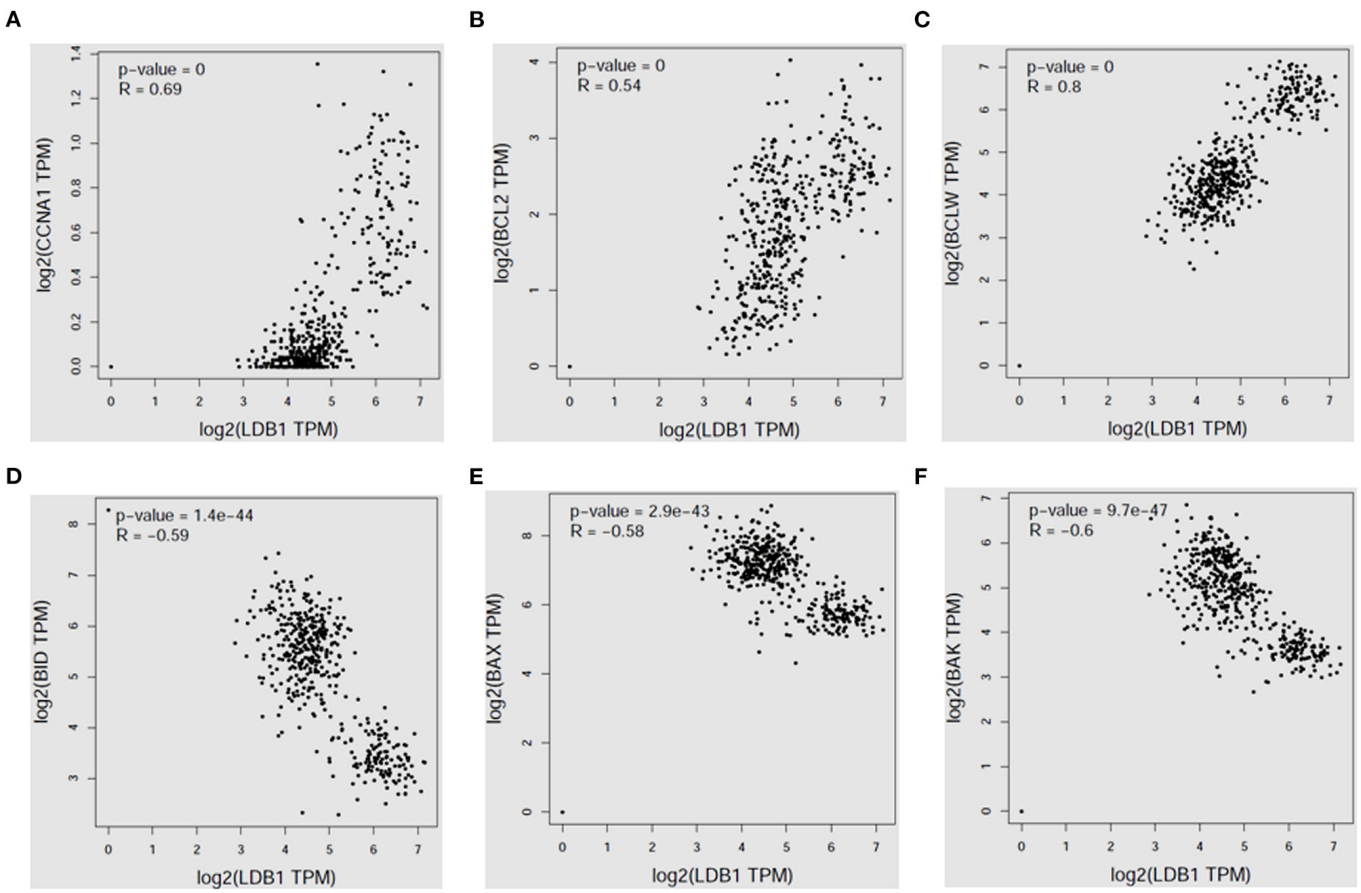
LDB1 expression is positively correlated with survival signals in colorectal cancer. The correlation analysis between LDB1 expression and CCNA1 **(A)**, BCL2 **(B)**, BCLW **(C)**, BID **(D)**, BAX **(E)** or BAK **(F)** in colon adenocarcinoma was analyzed by GEPIA database.

Then, the correlation analysis between LDB1 expression and survival related genes' expression in CRC was analyzed by the public cancer database GEPIA, and we found that LDB1 expression was positively correlated with CCNA1, BCL2 and BCLW (Figures 2A–C), but negatively correlated with the pro-apoptotic signals BID, BAX and BAK (Figures 2D–F).

**Figure 3:**
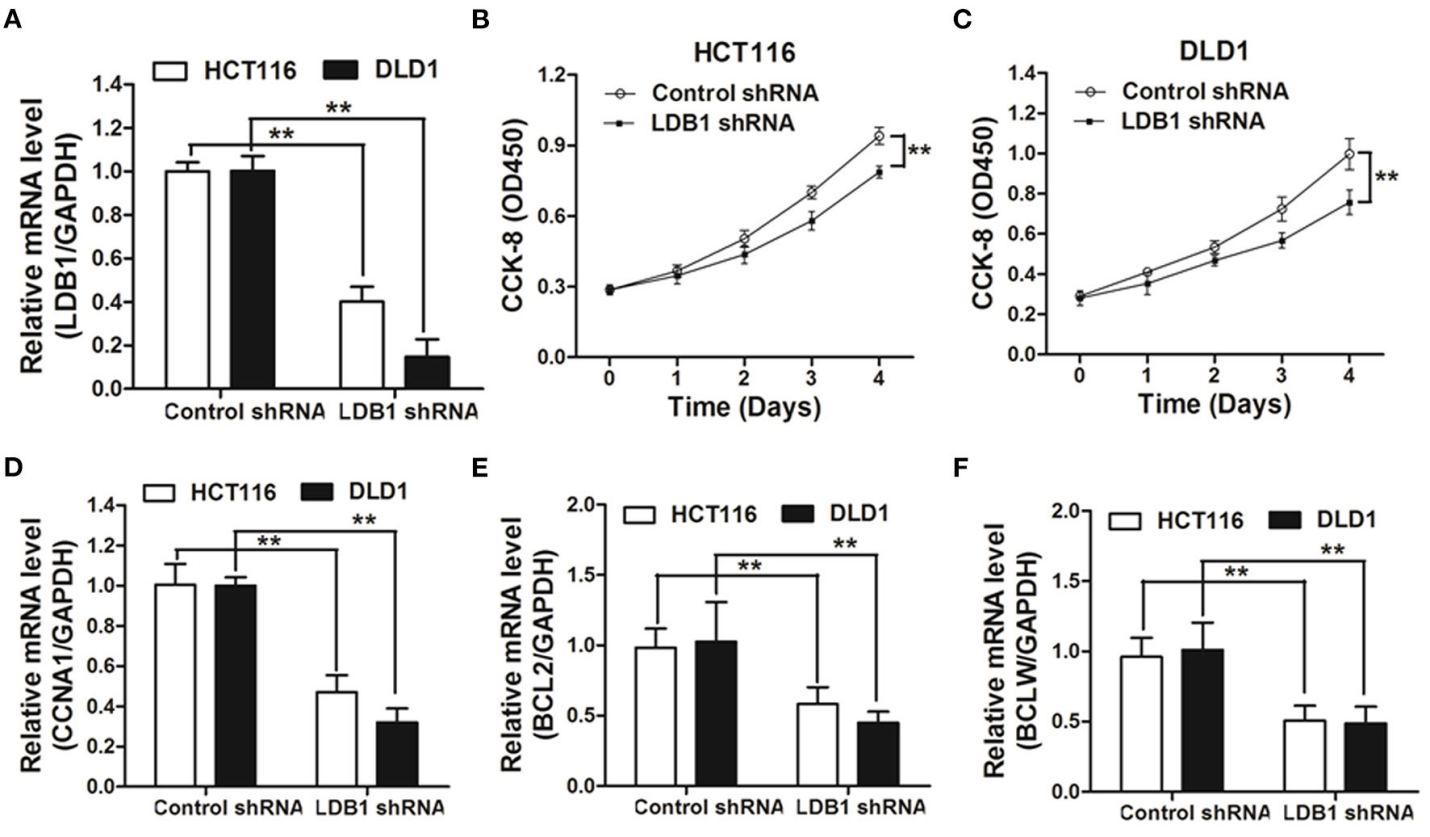
Knockdown of LDB1 inhibits cell proliferation in colorectal cancer. **(A)** The silencing effects of LDB1 shRNA in HCT116 and DLD1 cells were analyzed by qRT-PCR. **(B,C)** HCT116 **(B)** and DLD1 **(C)** cells infected with control or LDB1 shRNA-derived lentivirus were cultured for 0, 1, 2, 3 or 4 days, followed by CCK-8 assay. **(D-F)** HCT116 and DLD1 cells were infected with control or LDB1 shRNA-derived lentivirus for 3 days, and then cells were prepared for qRT-PCR analysis to detect the expression of CCNA1 **(D)**, BCL2 **(E)** and BCLW **(F)**. GAPDH was used as an internal control **P < 0.01.

To further assess the function of LDB1 in CRC, shRNAs were used to silence the expression of LDB1 in HCT116 and DLD1 cells. As shown in Figure 3A, qRT-PCR analysis showed that LDB1 was significantly silenced in both of HCT116 and DLD1 cells. Then, CRC cells infected with control or LDB1 shRNA-derived lentivirus were cultured for 0, 1, 2, 3 or 4 days, and the CCK-8 assay showed that silence of LDB1 could markedly inhibit cell growth in both of HCT116 (Figure 3B) and DLD1 cells (Figure 3C). Moreover, silence of LDB1 significantly inhibited the expression levels of CCNA1, BCL2 and BCLW, which were closely associated with cell growth (Figures 3D–F).

**Figure 4:**
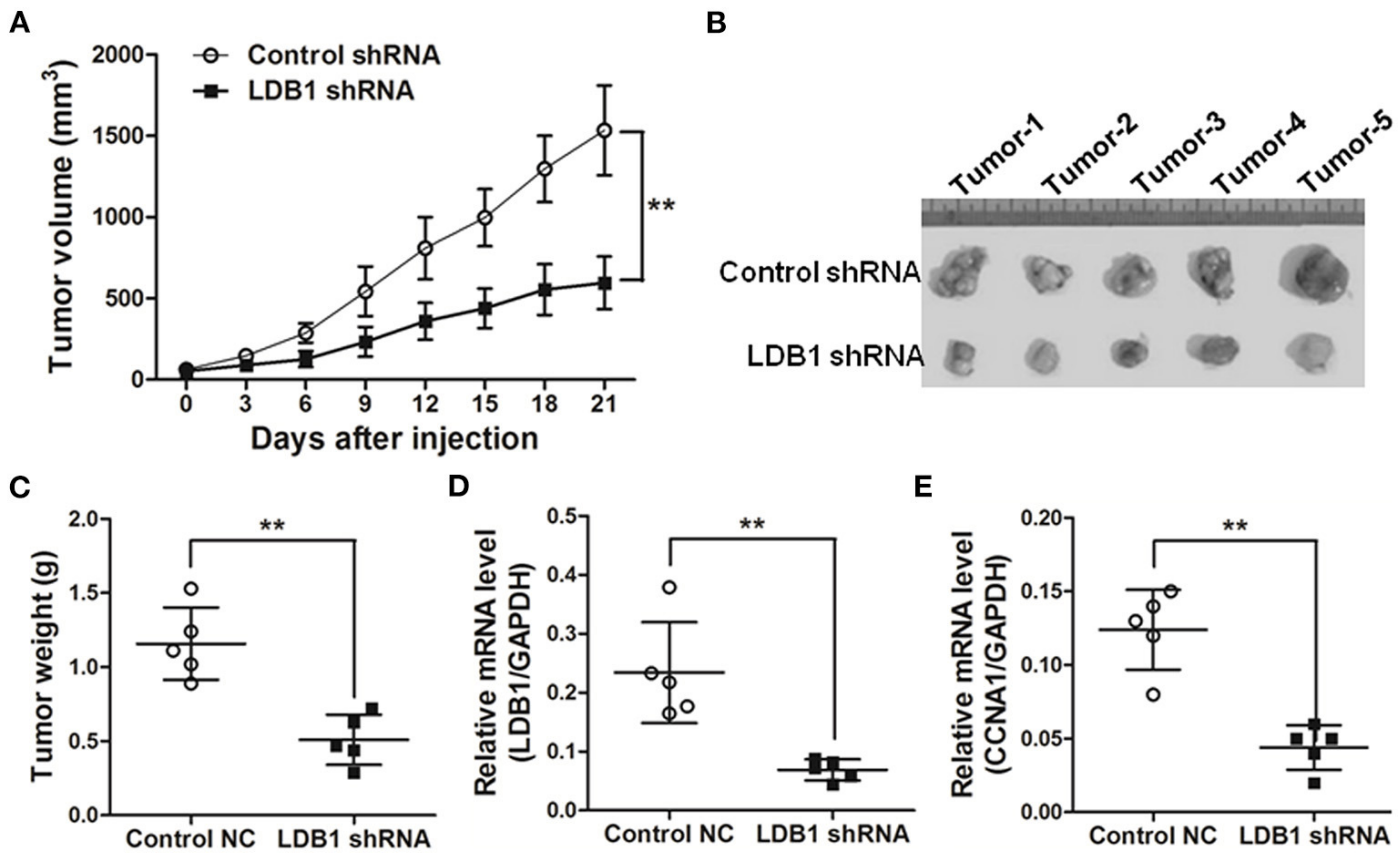
Knockdown of LDB1 inhibits tumor growth in nude mice. **(A)** The tumor growth curves. **(B,C)** At the end of the animal study, tumors were excised and taken photos **(B)**. And the tumors were also weighed **(C)**. **(D,E)**. Tumors were also prepared for qRT-PCR analysis to detect LDB1 **(D)** and CCNA1 **(E)** expression. GAPDH was used as an internal control **P < 0.01.

In order to verify the role of LDB1 in CRC more accurately, xenograft models were established. As shown in Figure 4A, the tumor growth curves showed that silence of LDB1 significantly suppressed the tumor growth of CRC. After the animal study, tumors were excised, taken photos and weighed, and we could clearly see that the tumor size in LDB1-silenced group was significantly smaller than the control (Figures 4B,C). The silenced efficacy of LDB1 shRNAs in tumors was also assessed (Figure 4D), and CCNA1 was also downregulated in LDB1 shRNA group (Figure 4E). Above results indicated that LDB1 silence inhibited CRC growth both *in vitro* and *in vivo*.

### LDB1 Is Associated With Oxaliplatin Sensitivity of Colorectal Cancer Cells

**Figure 5:**
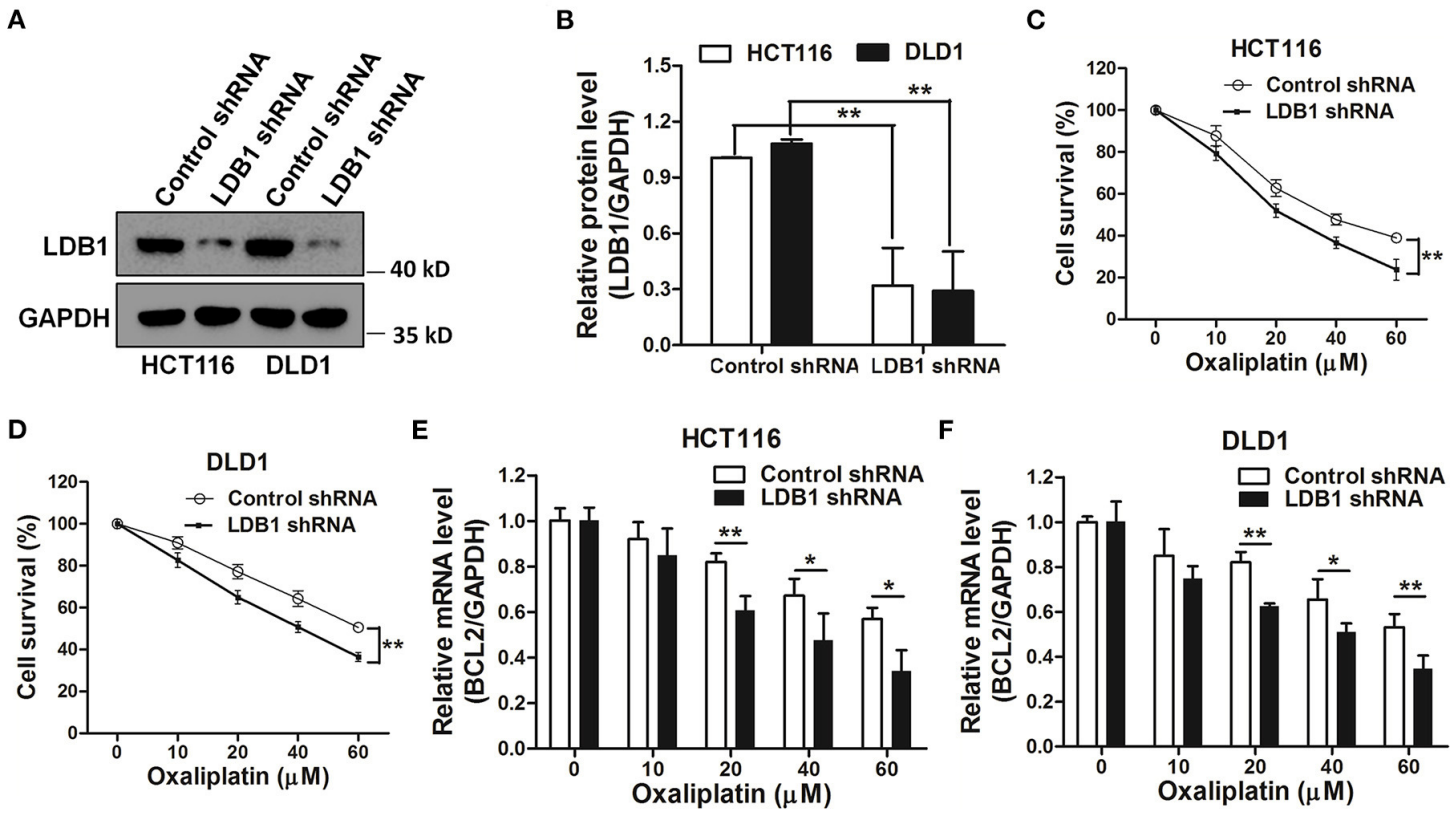
Knockdown of LDB1 sensitizes the drug sensitivity of oxaliplatin in colorectal cancer cells. **(A,B)** The silencing effects of LDB1 shRNA in HCT116 and DLD1 cells were analyzed by western blot (A), and the optical density analysis was also carried out **(B)**. **(C,D)** HCT116 **(C)** or DLD1 **(D)** cells infected with control or LDB1 shRNA-derived lentivirus were incubated with indicated concentrations of oxaliplatin for 48 h, followed by CCK-8 assay. **(E,F)** HCT116 (E) and DLD1 **(F)** cells infected with control or LDB1 shRNA-derived lentivirus were incubated with indicated concentrations of oxaliplatin for 48 h, followed by qRT-PCR to assess the expression of BCL2 *P < 0.05, **P < 0.01.

In order to further investigate the relationship between LDB1 and drug sensitivity in CRC cells, LDB1 was silenced by shRNAs. As shown in Figures 5A,B, the results of western blot showed that LDB1 was markedly silenced by shRNAs in both of HCT116 and DLD1 cells. Then, HCT116 and DLD1 cells infected with control or LDB1 shRNA-derived lentivirus were incubated with increasing concentrations of oxaliplatin for 48 h, and the CCK-8 assay showed that CRC cells with decreased LDB1 expression were more sensitive to oxaliplatin (Figures 5C,D). Additionally, the decrease of BCL2 in control group was higher than that in LDB1 shRNA group (Figures 5E,F). These results indicated that LDB1 was associated with oxaliplatin sensitivity of CRC cells.

### LDB1 Is Upregulated in Oxaliplatin-Resistant Colorectal Cancer Cells

**Figure 6:**
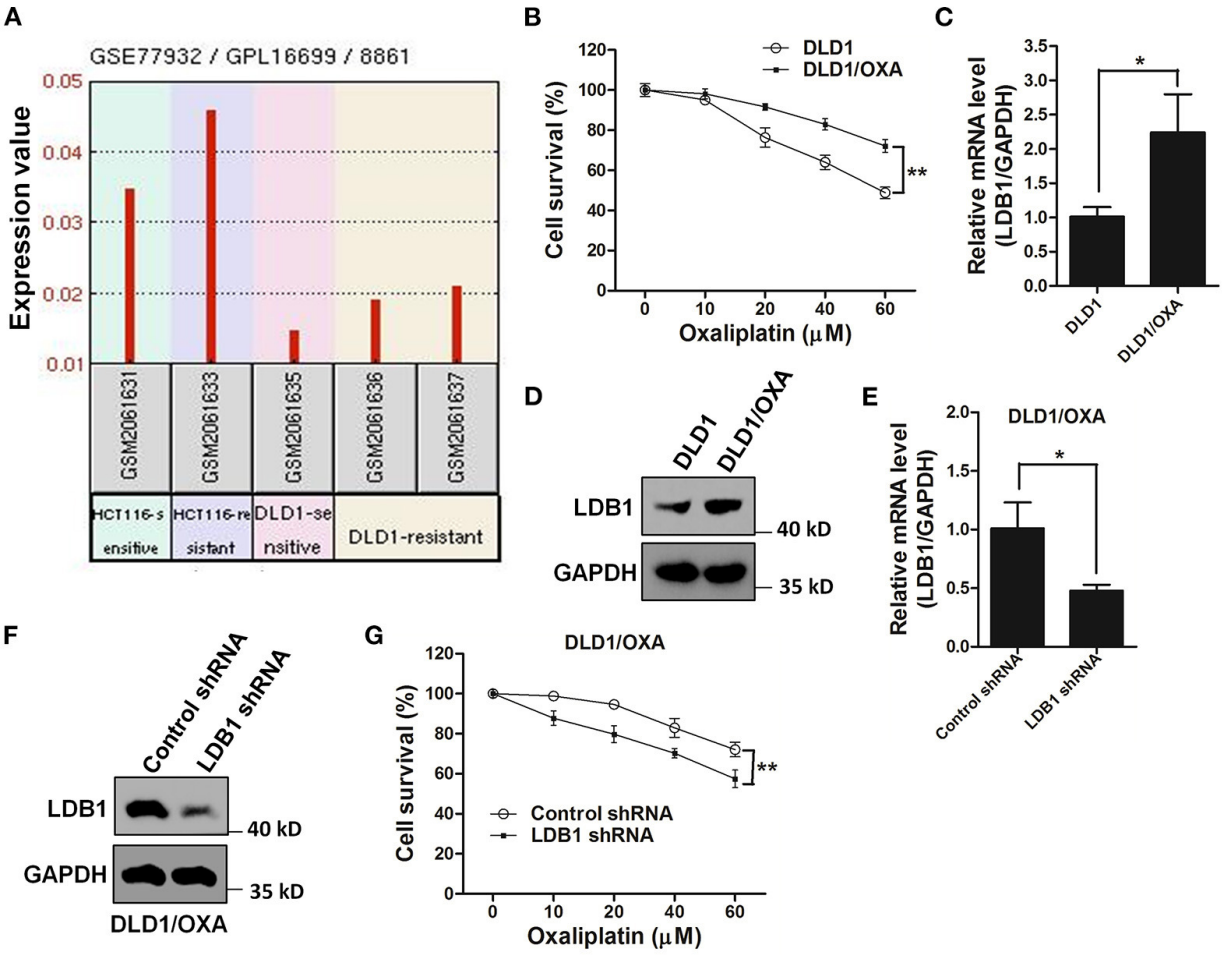
LDB1 is involved in the drug resistance of oxaliplatin in colorectal cancer cells. **(A)** The gene expression levels of LDB1 in oxaliplatin-sensitive HCT116, oxaliplatin-resistant HCT116 (HCT116/OXA), oxaliplatin-sensitive DLD1 and oxaliplatin-resistant DLD1 (DLD1/OXA) were obtained from GEO database (GEO accession: GSE77932). **(B)** DLD1 and DLD1/OXA cells were incubated with indicated concentrations of oxaliplatin for 48 h, followed by CCK-8 assay. **(C,D)** The expression levels of LDB1 in DLD1 and DLD1/OXA cells were analyzed by qRT-PCR **(C)** and western blot **(D)**. **(E,F)** The silencing effects of LDB1 shRNA in DLD1/OXA cells were analyzed by qRT-PCR **(E)** and western blot **(F)**. **(G)** DLD1/OXA cells infected with control or LDB1 shRNA-derived lentivirus were incubated with indicated concentrations of oxaliplatin for 48 h, followed by CCK-8 assay. *P < 0.05, **P < 0.01.

The GEO database showed that the expression of LDB1 was relatively increased in oxaliplatin-resistant cell lines (Figure 6A). Then, in order to further investigate the relationship between LDB1 and oxaliplatin resistance in CRC cells, an oxaliplatin-resistant cell line (DLD1/OXA) was established. CCK-8 assay showed that DLD1/OXA cells were not more sensitive to oxaliplatin than the wild-type cells (Figure 6B). QRT-PCR and western blot analysis also showed that LDB1 was markedly upregulated in DLD1/OXA cells compared with the wild-type cells (Figures 6C,D). Then, LDB1 was also silenced by shRNAs in DLD1/OXA cells (Figures 6E,F), and the silenced cells were also prepared for oxaliplatin treatment. As shown in Figure 6G, after silencing LDB1 in DLD1/OXA cells, the sensitivity of cells to oxaliplatin was significantly restored. These results further demonstrated the relationship between LDB1 and oxaliplatin resistance in CRC cells.

## Discussion

For patients with early CRC, surgery is the main strategy for the treatment; for patients with advanced CRC, chemotherapy is recommended (16). Among them, the combination of 5-FU with leucovorin and platinum chemotherapy has become the preferred treatment for CRC patients, and its overall survival rate is significantly higher than that of platinum-free chemotherapy (17). However, in recent years, it has been found that the phenomenon of chemotherapy resistance is more and more serious, and most of CRC patients with chemotherapeutic treatment within a year develop drug resistance (18).

As a cytotoxic drug, oxaliplatin can promote the apoptosis of tumor cells (19). When the apoptotic pathway is blocked, cells will develop drug resistance (20). Since the discovery of multidrug-resistance genes, researchers have conducted in-depth research on the mechanism of oxaliplatin resistance (21). Interestingly, our present study found that LDB1 may be a novel mediator for oxaliplatin resistance in CRC.

Our current results showed that silence of LDB1 could enhance the drug sensitivity of oxaliplatin in CRC cells, and our previous study also showed that overexpression of LDB1 could significantly reduce the drug sensitivity of 5-FU in CRC cells, which further indicated that LDB1 was closely involved in the chemosensitivity of CRC (13). What's more, an oxaliplatin-resistant CRC cell line was established to further evaluate the role of LDB1 in chemotherapy resistance, and LDB1 was found upregulated in oxaliplatin-resistant CRC cells. Moreover, silence of LDB1 could partly restore the sensitivity of cells to oxaliplatin in the oxaliplatin-resistant CRC cells. In addition, RNF38, an E3 for LDB1 or other substrates, has been reported to enhance 5-FU resistance in CRC, which further indicated that LDB1 may be an important mediator for drug resistance in CRC cells (22, 23).

Coincidentally, previous papers showed that overexpression of LDB1 could promote the proliferation of CRC cells, however, whether silenced LDB1 in turn affected the proliferation of CRC cells had not been reported (13, 24). In our present reports, we confirmed that silence of LDB1 significantly inhibited CRC cell growth in vitro, and the animal study also showed that knockdown of LDB1 markedly suppressed the tumor growth of CRC in vivo. In addition, LDB1 expression was found positively correlated with survival signals in CRC. These results above may explain why cells with high expression of LDB1 are more likely to develop drug resistance.

This paper just further confirms the roles of LDB1 in promoting cancer and inducing drug resistance in CRC, but how to exert these roles is still unclear, which will be further revealed in our future studies. In addition, we did not successfully establish a murine multi-drug resistant model to further confirm the mechanism of LDB1 in the drug resistance of CRC. In our future experiments, we will explore how to establish the corresponding model to further consolidate our current findings. In summary, our present study demonstrated a potential role of LDB1 in the growth and drug resistance of CRC cells. These results may provide a potential target for the clinical treatment of CRC in the future.

## Data Availability Statement

The original contributions presented in the study are included in the article/supplementary material, further inquiries can be directed to the corresponding authors.

## Ethics Statement

The studies involving human participants were reviewed and approved by the Review and Ethics Committee of The Affiliated Huaian No.1 People's Hospital of Nanjing Medical University. The patients/participants provided their written informed consent to participate in this study. The animal study was approved by the Review and Ethics Committee of The Affiliated Huaian No.1 People's Hospital of Nanjing Medical University.

## Author Contributions

MZ and ZH participated in the conception and design of the study. MZ, BJ, HZ, XW, and HG performed the experiments. MZ and ZH interpreted the data and produced the main document. All authors read and approved the final manuscript.

## Funding

This study was funded by the Science and Technology Project of Huaian (HAB202015), and the Fund for development of Science and Technology of Nanjing Medical University (NMUB2019350).

## Conflict of Interest

The authors declare that the research was conducted in the absence of any commercial or financial relationships that could be construed as a potential conflict of interest.

## Publisher's Note

All claims expressed in this article are solely those of the authors and do not necessarily represent those of their affiliated organizations, or those of the publisher, the editors and the reviewers. Any product that may be evaluated in this article, or claim that may be made by its manufacturer, is not guaranteed or endorsed by the publisher.

## References

[1] Siegel RL Miller KD Goding Sauer A Fedewa SA Butterly LF Anderson JC. Colorectal cancer statistics, 2020. CA Cancer J Clin. (2020) 70:145–64. 10.3322/caac.21601 32133645

[2] Kishore C Bhadra P. Current advancements and future perspectives of immunotherapy in colorectal cancer research. Eur J Pharmacol. (2021) 893:173819. 10.1016/j.ejphar.2020.173819 33347822

[3] Hu XH Niu WB Zhang JF Li BK Yu B Zhang ZY. Treatment strategies for colorectal cancer patients in tumor hospitals under the background of corona virus disease 2019. Zhonghua Wei Chang Wai Ke Za Zhi. (2020) 23:201–8. 10.3760/cma.j.cn.441530-20200217-00058 32192294

[4] Wu MM Zhang Z Tong CWS Yan VW Cho WCS To KKW. Repurposing of niclosamide as a STAT3 inhibitor to enhance the anticancer effect of chemotherapeutic drugs in treating colorectal cancer. Life Sci. (2020) 262:118522. 10.1016/j.lfs.2020.118522 33011217

[5] Luo X Teng QX Dong JY Yang DH Wang M Dessie W. Antimicrobial peptide reverses ABCB1-mediated chemotherapeutic drug resistance. Front Pharmacol. (2020) 11:1208. 10.3389/fphar.2020.01208 32903706 PMC7438908

[6] Köberle B Schoch S. Platinum complexes in colorectal cancer and other solid tumors. Cancers (Basel). (2021) 13:2073. 10.3390/cancers13092073 33922989 PMC8123298

[7] Biller LH Schrag D. Diagnosis and treatment of metastatic colorectal cancer: a review. JAMA. (2021) 325:669–85. 10.1001/jama.2021.0106 33591350

[8] Luo XJ Zhao Q Liu J Zheng JB Qiu MZ Ju HQ. Novel genetic and epigenetic biomarkers of prognostic and predictive significance in stage II/III colorectal cancer. Mol Ther. (2021) 29:587–96. 10.1016/j.ymthe.2020.12.017 33333293 PMC7854353

[9] Liu G Dean A. Enhancer long-range contacts: the multi-adaptor protein LDB1 is the tie that binds. Biochim Biophys Acta Gene Regul Mech. (2019) 1862:625–33. 10.1016/j.bbagrm.2019.04.003 31022553

[10] Li L Mitra A Cui K Zhao B Choi S Lee JY. Ldb1 is required for Lmo2 oncogene-induced thymocyte self-renewal and T-cell acute lymphoblastic leukemia. Blood. (2020) 135:2252–65. 10.1182/blood.2019000794 32181817 PMC7316212

[11] Layer JH Christy M Placek L Unutmaz D Guo Y Dave UP. LDB1 Enforces Stability on Direct and Indirect Oncoprotein Partners in Leukemia. Mol Cell Biol. (2020) 40:e00652–19 10.1128/MCB.00652-19 32229578 PMC7261719

[12] Simonik EA Cai Y Kimmelshue KN Brantley-Sieders DM Loomans HA Andl CD. LIM-Only Protein 4 (LMO4) and LIM Domain Binding Protein 1 (LDB1) Promote Growth and Metastasis of Human Head and Neck Cancer (LMO4 and LDB1 in Head and Neck Cancer). PLoS ONE. (2016) 11:e0164804. 10.1371/journal.pone.0164804 27780223 PMC5079595

[13] Huang Z Yang P Ge H Yang C Cai Y Chen Z. RING Finger Protein 38 Mediates LIM Domain Binding 1 Degradation and Regulates Cell Growth in Colorectal Cancer. Onco Targets Ther. (2020) 13:371–9. 10.2147/OTT.S234828 32021282 PMC6969705

[14] Kashiwagi E Izumi H Yasuniwa Y Baba R Doi Y Kidani A. Enhanced expression of nuclear factor I/B in oxaliplatin-resistant human cancer cell lines. Cancer Sci. (2011) 102:382–6. 10.1111/j.1349-7006.2010.01784.x 21087353

[15] Feng X Liu H Zhang Z Gu Y Qiu H He Z. Annexin A2 contributes to cisplatin resistance by activation of JNK-p53 pathway in non-small cell lung cancer cells. J Exp Clin Cancer Res. (2017) 36:123. 10.1186/s13046-017-0594-1 28886730 PMC5591524

[16] Tachimori A Yonemitsu K Fukui Y Tashima T Nishimura J Aomatsu N. Clinical significance of preoperative chemotherapy for advanced colorectal cancer. Gan To Kagaku Ryoho. (2020) 47:2021–3. 33468787

[17] Rottenberg S Disler C Perego P. The rediscovery of platinum-based cancer therapy. Nat Rev Cancer. (2021) 21:37–50. 10.1038/s41568-020-00308-y 33128031

[18] Lee GY Lee JS Son CG Lee NH. Combating drug resistance in colorectal cancer using herbal medicines. Chin J Integr Med. (2021) 27:551–60. 10.1007/s11655-020-3425-8 32740824

[19] Cao P Xia Y He W Zhang T Hong L Zheng P. Enhancement of oxaliplatin-induced colon cancer cell apoptosis by alantolactone, a natural product inducer of ROS. Int J Biol Sci. (2019) 15:1676–84. 10.7150/ijbs.35265 31360110 PMC6643222

[20] Lin J Song T Li C Mao W. GSK-3beta in DNA repair, apoptosis, and resistance of chemotherapy, radiotherapy of cancer. Biochim Biophys Acta Mol Cell Res. (2020) 1867:118659. 10.1016/j.bbamcr.2020.118659 31978503

[21] Nagourney RA Evans S Tran PH Nagourney AJ Sugarbaker PH. Colorectal cancer cells from patients treated with FOLFOX or CAPOX are resistant to oxaliplatin. Eur J Surg Oncol. (2021) 47:738–42. 10.1016/j.ejso.2020.09.017 33004272

[22] Long Y Zhao Q Huang Y. RNF38 enhances 5-Fluorouracil resistance in colorectal cancer by activating the Wnt pathway. Journal of BUON. (2021) 26:1246–51. 34564977

[23] Zhang J Wu H Yi B Zhou J Wei L Chen Y. RING finger protein 38 induces gastric cancer cell growth by decreasing the stability of the protein tyrosine phosphatase SHP-1. FEBS Lett. (2018) 592:3092–100. 10.1002/1873-3468.13225 30112836

[24] Garcia SA Swiersy A Radhakrishnan P Branchi V Kanth Nanduri L Gyorffy B. LDB1 overexpression is a negative prognostic factor in colorectal cancer. Oncotarget. (2016) 7:84258–70. 10.18632/oncotarget.12481 27713177 PMC5356660

